# 

**Published:** 1911-12-15

**Authors:** 


### ANNOUNCEMENTS.

Preliminary Programme of the Chicago Dental Society and Institute of Dental Pedagogics.

### MONDAY.

CHICAGO DENTAL SOCIETY. January 22nd, 1912. Manufacturer's Exhibit (All day.)

Monday Evening, 8 o'clock. "Comparative Dental Anatomy."

DR. WILLIAM BEBB.

### TUESDAY.

January 23rd, Clinic (All day.) College of Dentistry, University of Illinois.

Tuesday Evening, 8 o'clock. Paper—"Salivary Calculus."

DR. G. V. BLACK.

### WEDNESDAY.

INSTITUTE OF DENTAL PEDAGOGICS. January 24th, 9 o'clock. Address of Welcome.

THE MAYOR OF CHICAGO.

President's Address.

DR. DONALD M. GALLIE.

Report of Master of Exhibits. Report of Commission on Text Books.

Wednesday Afternoon, 2 to 5 o'clock. Paper—"The Teaching of Comparative Dental Anatomy."

DR. WILLIAM BEBB.

Wednesday Evening, 8 o'clock. Banquet. Address—(Prominent speaker of national reputation.)

THURSDAY.

January 25th, 9.30 to 11.30 A. M. Visit Northwestern University Dental School.

Luncheon, 11.30 to 12 o'clock. Automobiles to West Side.

Visit Chicago College of Dental Surgery, 1 to 3 o'clock. Visit College of Dentistry, University of Illinois, 3 to 5 o'clock

Thursday Evening, 8 o'clock. Paper—"The Teaching of Dental Histology."

DR. FRED B. NOYES.

FRIDAY.

January 26th, 9 o'clock. "The Teaching of Applied Physics and Chemistry."

DR. MARCUS L. WARD.

One Thirty o'clock. "The Teaching of Clinical Dental Pathology."

DR. H. T. SMITH.

Report of Commission on Nomenclature. Report of Dental Index Bureau.

